# Effect of inhaled salbutamol on whole-blood potassium concentrations in healthy cats

**DOI:** 10.1177/1098612X251320297

**Published:** 2025-04-11

**Authors:** Aina Salvà, Tristan Juette, Jo-Annie Letendre

**Affiliations:** 1 Department of Clinical Sciences, Ontario Veterinary College, University of Guelph, Guelph, ON, Canada; 2 Department of Clinical Sciences, Faculty of Veterinary Medicine, Université de Montréal, QC, Canada

**Keywords:** Potassium, hyperkalemia, salbutamol, treatment, albuterol

## Abstract

**Objectives:**

This study aimed to determine the effect of inhaled salbutamol on blood potassium concentrations in normokalemic cats, evaluate whether effects are dose dependent and assess whether it affects heart rate and blood glucose concentrations.

**Methods:**

The study was a prospective, open-label, two-way crossover trial. A total of 11 healthy cats were randomly assigned to two groups: one received a low dose of 100 µg salbutamol (Low group) and the other a high dose of 200 µg (High group). After a washout period of 15–21 days, the Low group received the high dose and the High group received the low dose. Blood potassium and glucose concentrations and heart rates were measured at baseline and 10, 20, 30, 45, 60, 90, 120, 150 and 180 mins after salbutamol administration.

**Results:**

Potassium concentrations significantly decreased over time after the administration of salbutamol in both groups (*P* <0.001). Salbutamol dose (µg/kg) and dose–time interaction had no significant effect on potassium concentration (*P* = 0.082 and *P* = 0.54, respectively). In the High group, mean potassium concentrations were significantly lower at 30–150 mins after salbutamol administration compared with baseline (*P* <0.011), and the mean decrease in potassium concentration from baseline to nadir was −0.69 ± 0.17 mmol/l. In the Low group, mean potassium concentrations were lower at 20–90 mins after salbutamol administration; however, these differences were not statistically significant (*P* >0.05). The administration of salbutamol did not appear to affect heart rate and blood glucose.

**Conclusions and relevance:**

In healthy cats, salbutamol administration led to a small and variable, non-dose-dependent decrease in potassium levels, suggesting that individual susceptibilities may affect the response to the potassium-lowering effects of salbutamol. The doses used in this study seemed safe. Further studies are needed to determine the optimal dosage of salbutamol and its effect on hyperkalemic cats.

## Introduction

Hyperkalemia (blood potassium concentration >5.5 mmol/l) is a frequent electrolyte disturbance in small animal emergency medicine.^1,2^ Oliguric or anuric acute kidney injury, urethral and ureteral obstructions, hypoadrenocorticism, ischemia–reperfusion injury, uroperitoneum and excessive intravenous (IV) infusion of potassium-containing fluids can result in hyperkalemia.^1,2^ Hyperkalemia can cause muscle weakness and electrocardiographic abnormalities, including peaked T-waves, widened QRS complexes and abnormal P-waves, and can lead to ventricular fibrillation and cardiac standstill.^3,4^ Fluid therapy, dextrose (with or without insulin), terbutaline and sodium bicarbonate are treatments commonly used to lower blood potassium concentration. Calcium gluconate is also used for its cardioprotective effects. Most therapeutic options aim to induce intracellular potassium displacement by stimulating the Na^+^/K^+^-ATPase pump or exchange between intracellular hydrogen ions and extracellular potassium ions.^1,3^

Salbutamol has been used for the treatment of acute, moderate-to-severe hyperkalemia in humans. Salbutamol is a short-acting β2-adrenergic receptor agonist commonly used for its bronchodilator effects.^5–7^ Its action in the treatment of hyperkalemia is primarily through direct β2-adrenergic stimulation of the Na^+^/K^+^-ATPase pump in skeletal muscles, which shifts potassium intracellularly, after inhaled or IV administration.^8,9^ Human clinical guidelines for managing hyperkalemia in adults recommend a salbutamol dose of 0.5 mg diluted in 100 ml dextrose 5% IV over 15 mins or 10–20 mg inhaled over 10 mins, to aid in the redistribution of potassium into cells. Effects are seen 6–8 mins (IV) or 30 mins (nebulized) after administration, peak at 30 mins (IV) or 90 mins (nebulized), and last 2–6 h.^9,10^ Salbutamol, particularly at higher therapeutic doses or with repeated administration, can lead to side effects such as tachycardia, hypokalemia and tremors.^8,11^ In overdose, hyperglycemia, lactic acidosis and cardiac arrhythmias have been described.^8^

Little is known about salbutamol’s potassium-lowering effect in animals. A recent prospective experimental study in healthy dogs demonstrated a significant reduction in serum potassium concentrations after the administration of inhaled salbutamol.^11^ To the authors’ knowledge, this is the first study assessing the effect of salbutamol on potassium concentrations in cats.

This study aimed to evaluate the effect of inhaled salbutamol on whole-blood potassium concentrations in a healthy cat population, to assess the impact of salbutamol dose (µg/kg) on the magnitude of potassium decrease and to determine the effect of salbutamol on glycemia and heart rate. We hypothesized that salbutamol would significantly decrease whole-blood potassium concentration and that the magnitude of potassium decrease would be dose dependent. Finally, we hypothesized that salbutamol would not induce tachycardia and hyperglycemia at the doses studied.

## Materials and methods

### Sample population

Healthy domestic cats belonging to the Centre Hospitalier Universitaire Vétérinaire, Montréal, teaching colony were eligible for inclusion if they were older than 1 year and weighed more than 3.5 kg. Cats were considered healthy if they had a normal physical examination and a normal basic metabolite panel (epoc Blood Analysis System; Siemens Healthineers), which were performed before each phase of the study protocol. They were excluded if they had an abnormal whole-blood potassium concentration (reference interval 3.5–5.6 mmol/l), any previously documented medical condition or if arrhythmias (brady- or tachyarrhythmias) were detected upon auscultation by a board-certified small animal criticalist. Cats were also excluded if they were receiving medication other than preventive medication. Animal use and procedures were approved by the Animal Care and Use Committee of the Université de Montréal (approval number 23-Rech-2205) under the standards of the Canadian Council on Animal Care.

### Study design

**Figure 1:**
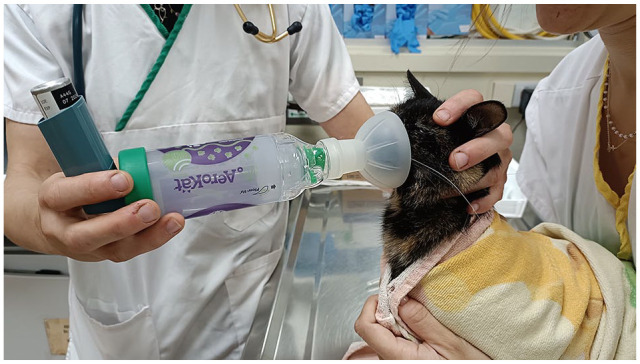
Salbutamol delivery via an AeroKat chamber attached to a tightly sealed silicone mask specifically designed for cats

The study was conducted with a prospective, open-label, two-way crossover design. The study was divided into two phases. For each phase, to facilitate blood sampling, a 20 or 22 G peripherally inserted central catheter (Drum Long Line Catheter; MILA International) was placed in the left medial saphenous vein. The catheter was placed under sedation with butorphanol (0.3–0.4 mg/kg, Dolorex 10 mg/ml; MSD Animal Health) and alfaxalone (2 mg/kg, Alfaxan Multidose 10 mg/ml; Zoetis) combined in the same syringe and administered intramuscularly. An Elizabethan collar was placed on all cats to avoid catheter damage or dislodgement. Sample collection began at least 1 h, but no more than 4 h, after catheter placement and recovery from sedation. Salbutamol sulfate (metered-dose inhaler, 100 µg/actuation, Ventolin; GlaxoSmithKline Pharmaceuticals) was delivered via an AeroKat chamber (Trudell Animal Health) attached to a cat-adapted tightly sealed silicone facemask (Figure 1). During phase 1, cats were randomly assigned to either the ‘low-dose’ group (Low), receiving 100 µg (one metered actuation), or the ‘high-dose’ group (High), receiving 200 µg (two metered actuations), using block randomization. The facemask was held in place for 12 breaths after inhaler actuation to ensure proper inhalation. In phase 2, executed 15–21 days later to follow the recovery guidelines set by the ethics committee, particularly regarding the volume of blood collected, cats that initially received the low dose were given the high dose, and vice versa. The dose in µg/kg was calculated for each cat in each phase. In both phases, heart rates were recorded after each blood sample acquisition at baseline (time [*t*] = 0; before delivery of inhaled salbutamol) and at 10, 20, 30, 45, 60, 90, 120, 150 and 180 mins after the 12th breath after inhaler actuation. At each time point, the catheter plug was removed (PRN Luer-Lock Adapter; BD) to reduce dead space and 1.5 ml of discard sample was collected to avoid sample dilution and contamination. Then, 0.4 ml of blood was obtained for analysis. The total volume collected at each phase was 10% or less of the cats’ blood volume. After sampling, the plug was replaced and the catheter was flushed with 1 ml of heparinized 0.9% saline. The blood sample was placed in a lithium heparin tube and analyzed within 2 mins with a blood gas analyzer (ABL90 FLEX PLUS analyzer; Radiometer). Blood potassium and glucose concentrations were recorded at each time point. Throughout the study period, automatic and regularly scheduled built-in quality control procedures were performed within the analyzer. Hypoglycemia and hyperglycemia were defined as blood glucose concentrations <3.8 mmol/l (68.4 mg/dl)^12^ and >7.9 mmol/l (142.2 mg/dl),^13^ respectively. Hypokalemia was defined as blood potassium concentrations <3.5 mmol/l.^1^ Azotemia was defined as creatinine >140 µmol/l.^14^ Bradycardia and tachycardia were defined as a heart rate <140 bpm and >240 bpm, respectively.^15^ Cats were under continuous observation and food was withheld throughout each phase. The catheter was removed after collection of the last sample.

### Statistical analysis

The effects of time, salbutamol dose (µg/kg) and dose–time interaction on potassium and glucose concentrations and heart rate were evaluated using linear mixed models (LMMs). To avoid potential pseudoreplication bias, the identity of individuals was included as a random factor in each LMM. The contribution of interpatient variability was assessed using the intraclass correlation coefficient (ICC). Normality of the residuals for each model was assessed through Shapiro–Wilk tests. Normally distributed variables are expressed as mean ± SD, whereas non-normally distributed variables are reported as median (range). Corrections were applied if residuals were not normally distributed. The results of each model were analyzed via a likelihood ratio test (LRT). If needed, post-hoc analysis was performed to compare the modalities two-by-two and determine which modalities differed from each other. Benjamini–Hochberg corrections were applied to the *P* values because of the multiple comparisons. The ICC was estimated for each model. *P* <0.05 was considered statistically significant. In the present study, the term ‘nadir’ describes the lowest value of a given variable.

## Results

### Sample population

In total, 11 cats fulfilled the inclusion criteria. Two cats participated in only one of the two phases because of baseline hypokalemia (3.4 mmol/l and 3.2 mmol/l, respectively). As a result, one cat received only the low dose and the other only the high dose. A third cat did not receive the high dose because of azotemia (creatinine 153 µmol/l) before the start of phase 2. A total of 10 cats were included in the Low group and nine cats in the High group. All individuals were adult domestic shorthair or domestic longhair cats. There were eight (73%) females and three (27%) males. One male was neutered and one female was spayed. The median body weight upon initiation of the study was 4.13 kg (range 3.69–5.13).

### Data collection

In the Low group, a clot obstructed one peripherally inserted central catheter, necessitating blood sampling via a peripheral IV catheter (Introcan Safety IV catheter, 20 G; B Braun) placed in the left medial saphenous vein. Discard sample, sample and heparinized saline flush volumes were respected as described above. Furthermore, in the Low group, no potassium and glucose results were obtained at *t* = 90 in one cat owing to an analytical error. For practical reasons, such as purring, restlessness and excessive movement, heart rate could not be obtained at every time point for some cats.

### Effect on potassium concentration

**Figure 2:**
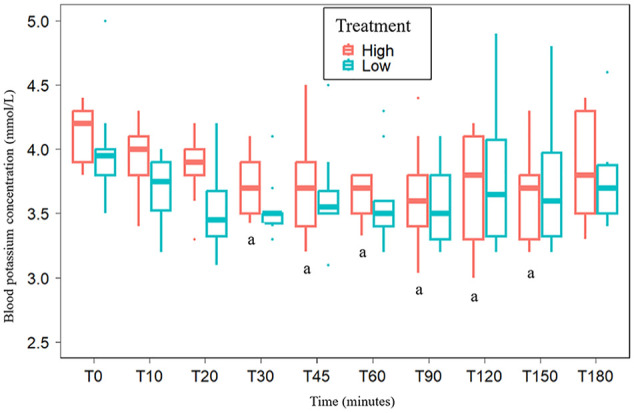
Box and whisker plot of blood potassium concentrations at various time points after inhaled administration of a low (100 µg) or high (200 µg) dose of salbutamol in 11 cats. a = mean potassium concentrations significantly different from baseline (T0) (*P* <0.05)

**Figure 3:**
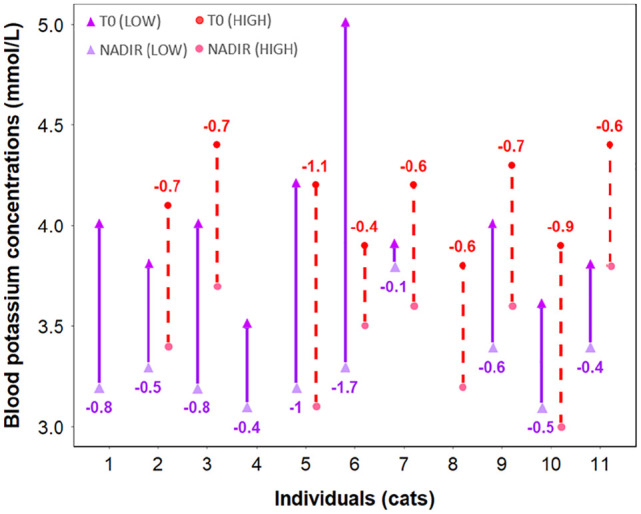
Scatter plot of blood potassium concentrations at baseline (T0) and at the lowest point (NADIR) after inhaled administration of a low (100 µg; purple) and high (200 µg; red) dose of salbutamol in each cat. The magnitude of decrease in potassium concentration (in mmol/l) for each cat is also noted. The mean maximal difference between baseline (T0) and minimal (NADIR) potassium concentration was 0.68 mmol/l and 0.7 mmol/l in the low-dose and high-dose groups, respectively, and was not significantly different (*P* = 0.082)

There was no significant difference in baseline potassium concentrations between treatment groups, with mean baseline potassium concentrations of 3.98 ± 0.41 mmol/l and 4.13 ± 0.22 mmol/l for the Low and High groups, respectively (*P* = 0.703). For both groups, potassium concentrations significantly decreased over time after administration of salbutamol (*P* <0.001). Salbutamol dose (µg/kg) and dose–time interaction had no significant effect on potassium concentration (*P* = 0.082 and *P* = 0.54, respectively). In the Low group, the mean potassium concentration was lower from *t* = 20 to *t* = 90 compared with baseline, but these differences were not statistically significant (*P* >0.05) (Figure 2). The potassium nadir of the Low group was 3.53 ± 0.23 mmol/l and occurred at *t* = 30. This value was not significantly lower than the mean baseline potassium concentration (*P* = 0.056). In the Low group, the change in potassium concentration between the baseline value and each time point was −0.39 ± 0.10 mmol/l. In this group, the mean change in potassium concentration from baseline to nadir for each individual was −0.68 ± 0.42 mmol/l, but this variation was not statistically significant. Cat 6 had the greatest decrease in potassium concentrations (–1.7 mmol/l) (Figure 3).

In the High group, mean potassium concentrations were significantly lower from *t* = 30 to *t* = 150 compared with baseline (*P* <0.011). The nadir of potassium in the High group was reached at *t* = 60 (3.63 ± 0.19 mmol/l), 90 (3.63 ± 0.45 mmol/l) and 150 (3.63 ± 0.36 mmol/l) (*P* = 0.001) (Figure 2). In the High group, the decrease in potassium concentration between the baseline value and each time point was −0.34 ± 0.08 mmol/l. In this group, the mean decrease in potassium concentration from baseline to the nadir of each cat was −0.69 ± 0.17 mmol/l. Cat 5 had the greatest decrease in potassium concentrations (–1.1 mmol/l) (Figure 3). The mean potassium concentration mildly increased towards baseline (3.83 ± 0.43 mmol/l) at *t* = 180 (*P* = 0.058). Hypokalemia was recorded at 26% (49/189) of time points (Low group: n = 29; High group: n = 20). In the Low group, the median potassium concentration of the time points associated with hypokalemia was 3.3 mmol/l (range 3.1–3.4) and hypokalemia occurred in nine cats. In the High group, the median potassium concentration of the time points associated with hypokalemia was 3.3 mmol/l (range 3.0–3.4) and hypokalemia occurred in four cats.

### Effect on glucose concentration

The median glucose concentrations at baseline were 4.85 mmol/l (range 4.4–8.1) for the Low group and 4.9 mmol/l (range 4.2–10.1) for the High group, and were not significantly different (*P* *=* 0.682*)*. For each group, glucose concentration did not follow a specific trend after salbutamol administration, and none of the blood glucose concentrations were significantly different from the baseline glucose concentration. Hyperglycemia was recorded at 9/189 (4.8%) time points (Low group: n = 8; High group: n = 1; not significantly different, *P* = 0.25). The median glucose concentration for the hyperglycemic values was 8.6 mmol/l (range 8.1–9.3) for the Low group and occurred in two cats. The first cat was hyperglycemic from *t* = 0 to *t* = 60, after which it had a normal blood glucose concentration. The second cat had a normal blood glucose concentration throughout the study period, except at *t* = 120 and *t* = 150. The glucose concentration reflective of the single hyperglycemia occurrence in the High group was 10.1 mmol/l. This cat was hyperglycemic at *t* = 0. Its blood glucose concentration normalized at *t* = 10 and remained within normal limits for the rest of the study period. No hypoglycemic episodes occurred.

### Effect on heart rate

The mean heart rate at baseline was 236 ± 29 bpm for the Low group and 232 ± 38 bpm for the High group, and these rates were not significantly different (*P* = 0.828). Similar to the observed changes in glucose concentration, heart rates in each group did not exhibit a specific trend after the administration of salbutamol. Tachycardia was recorded at 52/159 (33%) time points (Low group: n = 31; High group: n = 21; not significantly different, *P* = 0.66). In the Low group, the median heart rate for the tachycardic episodes was 268 bpm (range 244–288) and these episodes occurred in six cats. In the High group, the median heart rate for the tachycardic episodes was 260 bpm (range 244–280) and episodes occurred in four cats. No bradycardic events occurred.

## Discussion

Salbutamol administration in this population of healthy cats led to a decrease in blood potassium concentration over time. However, the magnitude of this decrease was small overall and highly variable from one individual to another. This study suggests that cats may have individual susceptibilities and variable responses to the potassium-lowering effects of salbutamol. Furthermore, the relatively small decrease in potassium concentrations after salbutamol administration raises the question of its clinical significance. In the present study, the magnitude of potassium decrease was not significantly correlated to the salbutamol dose (µg/kg). In the Low group, cats were given a dose in the range of 19.6–28 µg/kg salbutamol. In the High group, they were given a dose of 39.2–56 µg/kg. Despite lower potassium concentrations after salbutamol administration in the Low group, potassium concentrations never differed significantly from baseline. In the High group, blood potassium concentrations were significantly lower than baseline from *t* = 30 to *t* = 150. The administered doses were selected based on the therapeutic dosages recommended for the treatment of bronchoconstriction. The recommended dose of salbutamol for the treatment of feline asthma is one puff (10 µg), which may be repeated up to three times at intervals of 5–15 mins. For dogs, the recommended dose for treating bronchoconstriction is in the range of 20–50 µg/kg.^16^ Based on an estimated average weight of 4 kg for the cats included in the study, this corresponds to a dose of approximately 80–200 µg, according to the canine dosing guidelines. In this study, it is possible that in the Low group, the dose was too low to significantly affect potassium concentration.

A study on the effect of inhaled albuterol in healthy dogs demonstrated inter-individual variations in the potassium-lowering effect of albuterol.^11^ In this study, the mean potassium concentration of the groups at different times were used to determine the nadir time. However, when considering each cat individually, the time to reach the nadir varied considerably, as did the magnitude of the decrease between baseline and nadir. Similar individual variations in the time between salbutamol administration and its hypokalemic effect, the magnitude of the potassium decrease and the duration of the effect have also been described in human studies.^9,11,17^

As salbutamol was administered via inhalation, incomplete delivery of the intended dose due to variations in tolerance to restraint, respiratory rate or tidal volume may also have influenced the observed results. Indeed, blood salbutamol concentrations were not measured, bringing into question whether the dose administered was entirely inhaled and sufficient to impact on potassium concentrations. Assessing a wider range of salbutamol doses and comparing IV and inhaled administrations may help elucidate the impact of these factors. Furthermore, we studied the effect of a single dose of salbutamol over 3 h. Repeated salbutamol administration may lead to a prolonged and more pronounced decrease in potassium concentrations. Further studies are needed to investigate the effect of the dose and repeated administration of salbutamol on potassium concentration.

Cats were systematically sedated for the placement of peripherally inserted central catheters. The effect of sedative and induction agents on potassium concentrations is currently unknown. Difficulties in catheter handling and the total flush volume may have contributed to variations in potassium concentrations due to dilution or hemolysis. A study in cats found that blood samples collected through a vascular access port had slightly, but significantly, higher potassium concentrations compared with those taken directly from the jugular vein, likely due to some degree of hemolysis.^18^ In our study, it is possible that the potassium concentrations in blood samples obtained via a venous catheter were similarly affected by hemolysis. This represents a limitation of our study. However, given the need for multiple blood samples (20 in total per cat), the use of a sampling catheter was necessary for welfare reasons to minimize handling, restraint and stress.

Interestingly, catecholamines (ie, epinephrine [adrenaline], norepinephrine [noradrenaline]) may have potassium-lowering properties through a similar mechanism of action as salbutamol (membrane-bound Na^+^/K^+^-ATPase pump stimulation).^19^ In a previous human experimental study, IV administration of epinephrine at similar levels to those seen in acute illness induced hypokalemia to a similar extent to IV salbutamol administration. When administered together, salbutamol and epinephrine had a synergistic effect in lowering potassium concentrations.^10^ These findings suggest that individual variations in stress response to the experimental procedures might have contributed to the noted differences in the magnitude of potassium decrease.

No major side effects were detected in this study. Although mild hypokalemia was observed at over 25% of time points, no clinical signs of hypokalemia were observed Interestingly, more cats and more hypokalemia time points were observed in the Low group than in the High group, although the difference was not statistically significant. This could be explained by the lower, albeit non-significant baseline potassium concentration in the Low group. In this study, hyperglycemia was rare and no hypoglycemia episodes were observed. An experimental study in dogs showed a temporary, insignificant decrease in glucose concentration within the first 10 mins of inhaled albuterol administration. Hyperglycemia was observed at 21% of time points and no instances of hypoglycemia were reported.^11^ The frequency of hyperglycemia in our cat population (4.8% of time points) was lower than that described in the dog study. In this population, episodes of hyperglycemia seem to depend on the individual (only three cats showed glucose concentrations above normal, one of them even before salbutamol administration). Difficulties encountered during restraint and blood sampling, as well as stress, are therefore likely to be important contributing factors. Stress-induced hyperglycemia arises from increased insulin resistance, increased counter-regulatory hormone production (ie, catecholamines, cortisol) and decreased insulin secretion.^20,21^ Differences in response to handling and environmental stress may have interfered with the interpretation of the effect of salbutamol on blood glucose concentrations in this study. Human studies have reported hyperglycemia as a side effect of salbutamol.^8^ The low occurrence of hyperglycemia and the lack of clinically relevant changes in blood glucose concentrations after salbutamol administration in this study may be because of the use of relatively low doses compared with doses used in humans. Further investigation is required to assess the impact of different doses and repeated administration of salbutamol on blood glucose concentrations in cats.

Despite tachycardia being a reported side effect of salbutamol administration in humans, this study revealed no clinically significant impact of salbutamol on heart rate, and instances of observed tachycardia were likely induced by stress related to handling rather than by salbutamol administration. Handling the cats, whether for sample collection or heart rate measurement, may have caused stress, potentially affecting the heart rate. Ideally, the heart rate would have been measured using telemetry, thus avoiding the need to handle the cats. However, since the study was conducted on relatively young, healthy and active cats, it is unlikely that the electrodes would have stayed in place for the duration of the protocol or provided reliable results. Therefore, we chose to collect the blood first, as this procedure required a certain amount of preparation and collection time: approximately 30–60 s to remove the catheter plug, collect the blood, replace the plug and flush the catheter. This provided the cat with time to relax in the handler’s arms. Nevertheless, despite this precaution, stress may have still affected the cats’ heart rate and this remains a limitation of the study.

Despite its prospective nature, this study also has other limitations. The cats were healthy and normokalemic. It is crucial to recognize that the safety and potassium-lowering effect of inhaled salbutamol may vary in hyperkalemic, critically ill cats or cats with comorbid conditions. The impact of salbutamol on potassium concentration in hyperkalemic cats remains uncertain. In addition, there is a lack of clarity regarding the effectiveness of salbutamol compared with other treatments for hyperkalemia, as well as the potential for synergistic effects of combined treatments. This study shows that salbutamol may cause a decrease in potassium levels in healthy cats. However, the optimal dose and frequency of administration have not been established. Another limitation was the small sample size and multiple comparisons, which may have led to statistical type I and II errors. As mentioned earlier, the procedures used in this study may have caused temporary stress to the cats. Variations in individual stress levels may have affected blood potassium concentrations, making it difficult to assess the effects of salbutamol on potassium concentration. The use of the AeroKat inhalation chamber was a new experience for the cats in the study and could have caused stress for some. Finally, in some cats, purring, restlessness and stress-related erratic behavior prevented the heart rate being obtained at some time points. It is, however, unlikely that these missing data prevented the detection of tachycardia as a side effect of salbutamol.

## Conclusions

The administration of salbutamol in healthy cats resulted in a small and variable reduction in blood potassium concentration, implying that individual susceptibilities may influence the response to its potassium-lowering effects. The dose of salbutamol was not associated with the magnitude of the potassium-lowering effect. The administration of salbutamol at the doses used in this study appeared to be safe as there were no clinically relevant changes to blood glucose concentrations and heart rate. Two salbutamol actuations could be considered for the treatment of hyperkalemia in cats, but it is important to recognize that individual responses may vary. Future studies evaluating the efficacy of salbutamol as a treatment in hyperkalemic cats are needed.
